# How the loss of forest fauna undermines the achievement of the SDGs

**DOI:** 10.1007/s13280-021-01547-5

**Published:** 2021-04-06

**Authors:** Torsten Krause, Andrew Tilker

**Affiliations:** 1 Lund University Centre for Sustainability Studies, P.O. Box 170, 221-00 Lund, Sweden; 2 Global Wildlife Conservation, 500 Capital of Texas Hwy, Austin, TX 78746 USA; 3 Leibniz Institute for Zoo and Wildlife Research, Alfred-Kowalke-Straße 17, 10315 Berlin, Germany

**Keywords:** Carbon storage, Defaunation, Hunting, Nutrition, Security, Sustainable Development Goals, Tropical forest fauna, Well-being, Wild meat

## Abstract

The human-driven loss of biodiversity has numerous ecological, social, and economic impacts at the local and global levels, threatening important ecological functions and jeopardizing human well-being. In this perspective, we present an overview of how tropical defaunation—defined as the disappearance of fauna as a result of anthropogenic drivers such as hunting and habitat alteration in tropical forest ecosystems—is interlinked with four selected Sustainable Development Goals (SDGs). We discuss tropical defaunation related to nutrition and zero hunger (SDG 2), good health and well-being (SDG 3), climate action (SDG 13), and life on land (SDG 15). We propose a range of options on how to study defaunation in future research and how to address the ongoing tropical defaunation crisis, including but not limited to recent insights from policy, conservation management, and development practice.

## Introduction

The current loss of biological diversity is unprecedented (IPBES 2019). Species extinctions are several times the estimated background rate, and coincide with increasing human domination and alteration of the natural world (Ceballos et al. 2015). Of the approximately 128 500 species assessed by *The IUCN Red List of Threatened Species*, more than 28% are threatened with extinction (IUCN 2020). Despite a global rhetoric to address biodiversity loss, as well as numerous international and national-level political strategies for protecting biodiversity, there is no indication that the trajectory of global biodiversity declines will change in the near future (IPBES 2019).

Global biodiversity loss has been most acute in tropical regions globally, where species richness and endemism are particularly high (Barlow et al. 2018). Both deforestation and forest degradation have reduced the extent and quality of tropical forest habitats (Alroy 2017). However, while deforestation and forest degradation have received considerable political attention (UNFCCC 2016), a third “de”—defaunation, a term that refers to the disappearance of fauna as a result of anthropogenic drivers such as hunting and habitat alteration (Dirzo et al. 2014)—has been largely overlooked within forest conservation policies and forest protection mechanisms such as REDD+ (Reducing Emissions from Deforestation and forest Degradation), and is routinely left out of forest governance strategies (Krause and Nielsen 2019). This omission is detrimental insofar as defaunation has significant negative effects on tropical forest ecosystems, which in turn impacts biodiversity in these systems, as well as numerous socio-economic and cultural impacts that affect human well being.

In this perspective we link tropical defaunation to four of the 17 Sustainable Development Goals (SDGs) that were adopted by the UN Member States in 2015 to set out a 15-year plan for delivering these goals by 2030. We selected SDG 2 (nutrition and zero hunger), SDG 3 (good health and well-being), SDG 13 (climate action), and SDG 15 (life on land), because of the immediate linkages with tropical defaunation. For each of these four SDGs, we review how defaunation affects and potentially undermines these goals. We then discuss how to better study defaunation, with a focus on strengthening interdisciplinary approaches, and propose solutions to address the ongoing defaunation crisis within the context of the SDGs.

## Why does defaunation matter?

### Nutrition and zero hunger (SDG 2)

Wild meat can be an important source of nutrition for households living within and in close proximity to tropical forests. Recent studies provide insights into the linkages between SDG 2 and the loss of forest fauna, indicating that food security and several targets under SDG 2 are directly (e.g., target 2.1, 2.2) and indirectly (e.g., target 2.3, 2.4) related to defaunation (Rowland et al. 2017; Sunderland and Vasquez 2020).

A global comparative analysis conducted for 12 months between 2004 and 2010 across 37 sites in 25 tropical forest countries found that wild meat is an important contributor to diets for households living near forests (Rowland et al. 2017). Another study carried out by Nielsen et al. (2018) examined the role of wild meat in 7978 households in 24 countries in the Global South, concluding that reliance on wild meat is highest for poorer households. A regional study carried out in 2012 and 2013 by Sarti et al. (2015) in the Amazon’s tri-border region of Colombia, Peru and Brazil found that in urban households that consume wild meat it made up more than 30% of daily caloric intake, 72% of consumed protein, 77% of iron, and provided valuable macronutrients. In rural northeastern Madagascar, Golden et al. (2011) revealed how the consumption of wildlife was associated with significantly higher hemoglobin concentrations in preadolescent children. Friant et al. (2020) conducted a study in six forest communities bordering Nigeria’s Cross River National Park, showing that wild meat consumption was significantly associated with relatively higher household food security status. These studies indicate that for local populations that depend on wild animals for sustenance, including many indigenous communities, overexploitation of forest fauna destabilizes an important resource base (Sarti et al. 2015; Nielsen et al. 2018).

These studies provide evidence of the importance of wild meat and indicate that defaunation has implications for ending hunger and ensuring access to safe, nutritious and sufficient food throughout the year (target 2.1) and ending malnutrition for children under 5 years of age (2.2). Thus, a decline or disappearance of wild animals negatively affects human dietary quality, especially where there is limited access to markets or where people are poor (Rowland et al. 2017). Yet, in the absence of viable food alternatives and sources of protein, the necessity to obtain wild meat for consumption also drives unsustainable hunting and contributes to defaunation (Dobson et al. 2019).

Defaunation has indirect consequences on SDG 2 target 2.3—to double the agricultural productivity and incomes of small-scale food producers, and target 2.4—to ensure sustainable food production systems and implement resilient agricultural practices that increase productivity and production that help maintain ecosystems. Defaunation indirectly impacts flora communities in tropical forests, thereby impacting non-timber forest products (for example, wild fruit, nuts, rattan and bamboo) that are utilized by people (Rowland et al. 2017). The limited research that has been conducted on this topic suggests that such indirect effects could be significant. For example, because declines in larger vertebrates can reduce seed dispersal (Harrison et al. 2013) and defaunated forests may have less fruiting trees than forests with more intact faunal communities. In one example, Effiom et al. (2013), working in a highly defaunated forest in Nigeria, showed that declines of seed dispersing primates caused fundamental changes in fruiting tree densities, highlighting the transforming effects of hunting on fruiting trees. Furthermore, defaunation can lead to the breakdown of other plant–animal interaction beneficial to humans, such as pollination (Dirzo et al. 2014). Globally, approximately 87% of all flowering plants are pollinated by animals, and while the majority of pollinators are insects (Ollerton et al. 2011), an estimated 10% of described bird and 6% of described mammal species also act as pollinators (Regan et al. 2015). There is no question that declines in vertebrate pollinators can impact fruiting trees beneficial to humans. For instance, Aziz et al. (2017) showed that island flying foxes (*Pteropus hypomelanus*) are crucial for pollination of durian (*Durio zibethinus*), and discussed the impacts that declines in flying fox populations would have on this economically important fruiting tree. Further research is needed to understand the full effects of defaunation on pollination services for non-timber forest products for example wild edible plants, fruits and nuts that are important food resources for people (Regan et al. 2015).

### Good health and well-being (SDG 3)

Two SDG 3 targets relate directly to defaunation: target 3.3—to end the epidemics of neglected tropical diseases, and target 3.4—to promote mental health and well-being.

Hunter–wildlife interactions and wild meat consumption is of global public health importance because wild animals are hosts and transmitters of numerous deleterious—and potentially fatal—zoonotic diseases (Olival and Hayman 2014). Several major viral outbreaks—including SARS-CoV, Ebola, HIV and now SARS-CoV-2—have been linked to wildlife and its consumption in tropical areas (Wolfe et al. 2005; Andersen et al. 2020). For instance, pathogen spillover to human populations has been discussed within the context of hunting of African primates (Olivero et al. 2017) and the consumption of small carnivores in Asia (Bell et al. 2004). There is mounting evidence that the human-driven loss of tropical forest fauna can significantly increase dispersal of host, parasite, and vector species, thus enabling greater frequency of infectious disease outbreaks (Rogalski et al. 2017). Defaunation may also impact disease risk in less-obvious, indirect ways. One recent study, for example, found that defaunation-induced changes in trophic cascades can increase rodent densities and therefore affect the spread and prevalence of tick-borne pathogens (Young Hillary et al. 2017).

Wildlife has numerous impacts on human health and well-being, for instance its contribution to traditional medicines around the world. There is also increasing recognition in medicinal circles that traditional “zootherapy” (the use of living animals for medical treatment/animal assisted therapy), or the use of animal-based remedies, may have wider implications for modern medicine (Alves and Policarpo 2018). However, here it should also be pointed out that the demand for wildlife-based medicine—many of which have unfounded or dubious medical benefits—also represents a major and direct threat for numerous species around the world (Nature 2019). Thus, the associations between wildlife and human health are complex, and often depend on specific local or cultural contexts.

In addition to having material use, wild animals are culturally significant, feature prominently in belief systems, and serve as sources of inspiration in art and literature (Alves and Barboza 2018). Many indigenous cultures ascribe to animistic world views (Venz 2017), and the loss of tropical forest fauna potentially undermines core cultural identities for indigenous peoples. Furthermore, countries often have animals as national symbols, which represent important components of national cultural identity (Hammerschlag and Gallagher 2017). Nonetheless, Hammerschlag and Gallagher (2017) found that only 16% of these symbols are nationally protected and only 50% receive international trade restrictions, highlighting the fact that even prominent flagship species with cultural significance are not immune to overexploitation. From an intergenerational forward-looking perspective, the ongoing tropical biodiversity loss and widespread decline of animal populations will affect how individuals and societies as a whole will see their world in the future. There is a risk that people will become used to faunally impoverished ecosystems, both in the tropics and beyond. This so-called “shifting baselines syndrome” (Papworth et al. 2009) has implications for future generations, which may perceive the current baseline as normal, and therefore fail to realize the cultural changes that have already occurred as a result of defaunation.

### Climate action (SDG 13)

Defaunation plays a particular role for climate action, especially the SDG 13 target 13.1—strengthening resilience and adaptive capacity to climate-related hazards and natural disasters in all countries, and target 13.2—integrating climate change measures into national policies, strategies and planning.

Forests are the world’s most important terrestrial carbon sink, and maintaining and protecting healthy forest ecosystems is critical to mitigating climate change (Watson et al. 2018). Intact forest ecosystem functions and faunal communities play a crucial role in this process, because of the multitude of ecological interactions between plants and animals. The removal of forest fauna, in particular large-bodied vertebrates (in particular mammal and bird species), degrades these interactions and, as a result, can precipitate the breakdown of numerous ecological functions including those that impact carbon storage capacities (Dirzo et al. 2014; Bello et al. 2015; Benítez-López et al. 2017; Berzaghi et al. 2018). For example, large vertebrates are often important seed dispersers in tropical forests (Corlett 2017), and studies have shown that faunal declines can reduce seed dispersal (Effiom et al. 2013; Harrison et al. 2013), leading to fundamental shifts in tree species composition and forest biomass, which ultimately impact global carbon cycles (Bello et al. 2015; Brodie 2018; Berzaghi et al. 2019). Recent studies have shed light on this complex relationship between defaunation and declines in carbon storage within tropical forests. For instance, Osuri et al. (2016) used a tropical faunal dataset and simulations to show that declines in large-seeded animal-dispersed trees impacted wood volume in tropical forests, resulting in estimated carbon storage declines of up to 12%. Similarly, Bello et al. (2015) found that the loss of larger mammal and bird species in the Brazilian Atlantic Forest region reduced dispersal of large-seeded trees—many of which tend to have high wood density and larger stature, and therefore high carbon storage capacities. This, in turn, has the potential to cause significant erosion of tropical forest carbon storage capacities.

At the global scale, it is likely that defaunation therefore poses an indirect, but significant, threat to efforts to address climate change (Brodie and Gibbs 2009). Nonetheless, despite the increasing evidence of biodiversity-carbon-climate linkages (Thompson et al. 2012) global forest governance, including proposals that aim to curb deforestation and forest degradation and aim to foster more sustainable forest management (e.g., REDD+), largely fail to address forest fauna loss (Krause and Nielsen 2019).

### Life on land (SDG 15)

The tropical defaunation crisis is largely driven by habitat loss and degradation, and unsustainable wildlife exploitation. The loss of fauna from the world’s tropical forests is directly connected to the future ability to protect and sustainably use terrestrial forest ecosystems and their biodiversity. In the context of SDG 15, defaunation is an overarching threat for achieving its targets. Some of these targets are already likely to be missed, foremost target 15.2—by 2020, promoting the implementation of sustainable management of all types of forests, halt deforestation, restore degraded forests and substantially increase afforestation and reforestation globally, and target 15.5—taking urgent and significant action to reduce the degradation of natural habitats, halt the loss of biodiversity and, by 2020, protect and prevent the extinction of threatened species.

Tropical forests are increasingly empty of native fauna and species declines are ongoing and widespread. A recent meta-analysis by Benítez-López et al. (2017) with data from 176 studies published between 1970 and 2015, estimated that tropical bird and mammals had declined in abundance by 58% and 83%, respectively. A follow-up study estimated that approximately 47% of the global tropical forest area, or approximately 14 million km^2^, have been defaunated (Benítez-López et al. 2019). Not surprisingly, declines of this magnitude have pushed numerous vertebrate species to the brink of extinction. For example, in the tropical forests of Southeast Asia, hunting-induced defaunation has already contributed to the global extinction of two large ungulates, the kouprey (*Bos sauveli*) and Schomburgk’s deer (*Rucervus schomburgki*) (Duckworth et al. 2015; Timmins et al. 2016).

In addition to the direct threats and the documented decline of tropical forest fauna, defaunation has been linked to a number of indirect effects on tropical ecosystems. These indirect effects cause biodiversity declines and ecosystem degradation, further undermining SDG 15 and in particular target 15.2 and 15.5. Defaunation perturbs complex ecological relationships and has the potential to change evolutionary trajectories (Galetti et al. 2018; Emer et al. 2019). In one recent study, Carvalho et al. (2016) found microevolutionary changes in palms following the local extinction of avian frugivores. The exact evolutionary consequences of faunal loss in tropical ecosystems over longer time periods are difficult to predict, but there is little doubt that present-day defaunation will profoundly alter the remaining tropical forest ecosystems that future generations will inherit.

## Addressing defaunation in the context of the SDGs

**Fig. 1:**
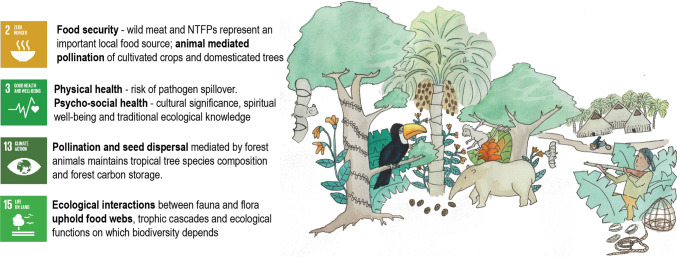
A summary of the interlinkages between defaunation and the SDGs. *Source* own illustration

Defaunation is intrinsically linked to numerous aspects of the SDGs and their specific targets. As a result, it is imperative that more attention is given to understanding how defaunation undermines these SDGs and the respective targets, and to develop strategies to preventing further loss of fauna from tropical ecosystems within their overarching context (Fig. 1).

### The need for more interdisciplinary research on defaunation

The consequences of the tropical defaunation crisis are uniquely complex and far-reaching: the loss of tropical forest fauna has countless ecological, evolutionary, socio-economic, and cultural repercussions, and undermines the achievement of the SDGs, in particular but not limited to SDGs 2, 3, 13 and 15. From a scholarly perspective, addressing the tropical defaunation crisis therefore requires a multi-faceted, interdisciplinary approach. To date, much defaunation research has occurred within traditional academic silos, with a strong focus on the ecological aspects of faunal loss. Such research is important, but ultimately needs to be integrated with other approaches if stakeholders across all levels are to have the information necessary to develop effective mitigation strategies and more sustainable tropical forest management and conservation practices.

Within this context, it is important for scholars studying tropical forest biodiversity to recognize that forests are inherently social–ecological systems, where biodiversity is inextricably linked to cultural diversity (Pretty et al. 2009). Defaunation is first and foremost a human-driven process and any research must therefore include a social and cultural dimension to understand how forest users and hunters make decisions and what local norms and management rules exist that are beneficial for sustainable management (Berkes 2018). Herein, it is particularly important for researchers to work with local experts, oftentimes local and indigenous hunters, to collect data that can answer scientific questions at the social–ecological nexus (Berkes 2017). Where unsustainable hunting is driven by large-scale commercialized wild meat or medicinal trades, it is important to understand the social norms that support these behaviors (Morsello et al. 2015).

### Designing holistic and place-based conservation approaches

Mitigating and reversing tropical defaunation is highly dependent upon local socio-economic and cultural contexts, as well as norms, institutions, and governance structures. Even within a single site, mechanisms for mitigating defaunation will likely evolve over time in response to ever-changing local conditions and external pressures. Nonetheless, developing effective solutions to the tropical defaunation crisis will be helped by approaching this issue within the SDG framework and making the interlinkages more visible; such an approach will help to connect local and regional defaunation of tropical forests to higher-level, long-term objectives associated with global well-being, and as a way to promote a more interdisciplinary approach that acknowledges its diverse consequences.

First of all, effective site-based protection is important considering that much of the world’s tropical biodiversity occurs in forest areas that have some degree of protected status. The question of who is best placed to manage wildlife populations, however, can be a complicated issue. Most traditional conservation strategies have relied on state-managed protected areas to conserve forest fauna. In situations where protected area governance structures are strong, with appropriate levels of economic support and institutional capacities, this approach can work well. India has shown that, with an effective protected area network, large mammals can thrive, even in areas that are surrounded by high human population densities (Reddy et al. 2016). Thus, successful site-based conservation approaches may mitigate or even prevent defaunation in tropical forests. In the short-term, this helps maintain healthy tropical forest ecosystems (SDG 15, targets 15.1, 15.2) and protect the threatened species that they harbor (target 15.5). In the long-term, the preservation of healthy forest systems within protected area networks, with intact faunal communities, is essential for strengthening climate resilience (SDG 13, target 13.1). Nonetheless, the darker side of strictly protected areas where conservation is sometimes enforced through militarization can, in the absence of appropriate oversight, lead to human rights violations and forced displacements, which highlight the need to balance effective conservation with equity and justice issues, particularly for local populations who primarily bear the cost of conservation measures (Verweijen 2020).

Despite some notable successes, there are numerous challenges for implementing and managing protected areas, particularly in tropical countries with insufficient resources, low levels of government support, and high levels of corruption. International cooperation and financing for biodiversity protection, as stipulated in the SDG 15 targets 15.2, 15.a and 15.b, is certainly crucial to achieve this, but is not enough to ensure equitable and effective outcomes when the underlying drivers of unsustainable use of natural resources from these areas are not addressed. In situations in which local communities retain some level of ownership of forest resources and are the primary users of wildlife, they have to be empowered to sustainably manage forest fauna as a means to ensure its long-term conservation. The fact that local communities often have a vested long-term interest in sustainably managed game populations can provide conservationists with a unique opportunity to use local hunters as potential allies to protect forest areas (Harrison et al. 2016; Ponta et al. 2019). Moreover, community-managed conservation approaches and indigenous owned lands have been shown in some cases to be more effective for ecosystem protection than standard state-run protected area systems (Garnett et al. 2018). Local community-based conservation approaches therefore have significant potential to maintain healthy tropical forest ecosystems, which not only contributes to the preservation of terrestrial biodiversity (SDG 15) and promotion of long-term carbon resilience (SDG 13), but may be particularly relevant to providing year-round access to safe and nutritious food (SDG 2, target 2.1) and ending malnutrition (target 2.2). Overall, there is no universal solution to effective site-based management, and conservation programs ultimately need to be tailored to local contexts, keeping in mind social and cultural factors as well as justice issues (Ruiz-Mallén et al. 2015).

### Recognizing and strengthening traditional ecological knowledge

For any forest governance mechanism and conservation effort to be fair and equitable, it is crucial to recognize the traditional knowledge, and conservation and management practices, of indigenous and local communities (Fui et al. 2012; Oteng-Yeboah et al. 2012; Pinedo-Vasquez et al. 2012). Traditional knowledge has typically (and historically) been undervalued and suppressed in recent centuries, but it still exists in some regions, even despite the ongoing process of erosion of biological diversity and supportive cultural beliefs (Aswani et al. 2018). Cultural values, local traditions, and religious beliefs can have positive and negative consequences for wildlife (Alves et al. 2012). Many indigenous and local societies, for instance the Kichwa in the Ecuadorian Amazon region, place important cultural or social values on the consumption and use of wildlife (Sirén 2012). This is exemplary of the wealth of traditional ecological knowledge and local cultural norms and traditional management rules that exists across the world’s tropical forest regions (Fui et al. 2012; Oteng-Yeboah et al. 2012; Pinedo-Vasquez et al. 2012). These norms and management rules that have developed over centuries can support a more sustainable use of forest resources and protect species, but they can also be a threat for specific species. In Madagascar, local villagers kill aye-aye (*Daubentonia madagascariensis*, Endangered) whenever it is encountered based on cultural taboos (Simons and Meyers 2001), while in Vietnam wild meat is consumed as a status symbol denoting wealth and generosity (Drury 2011). Other cultural traditions may support wildlife conservation. In some parts of Southeast Asia, “sacred forests” and some tree species of cultural importance are locally protected because they are associated with spirits of deities and ancestors (Fui et al. 2012). Some ethnic minority groups in Northeast India consider it taboo to hunt elephants (Velho and Laurance 2013), whereas Creole people in Belize refrain from hunting some primates (Jones and Young 2004). In general, these complex cultural norms need to be better understood to improve effective biodiversity conservation policies.

Researchers and practitioners working with conservation issues must understand that there are numerous cultural beliefs surrounding wildlife use, which are often neither static or unmalleable. It may be possible, for instance, to encourage taboos favorable for a more sustainable use of wildlife, and discourage the purposeful killing of species that are considered evil or bad luck (Golden and Comaroff 2015). This can be achieved through awareness campaigns and projects that built on social engagement, trust and local bottom-up processes. Incorporating these behavioral approaches into larger conservation strategies may ultimately help to foster local guardianship of wildlife resources, which could directly benefit the protection of threatened species (target 15.5). However, it should also be noted that traditional cultural beliefs, beneficial or not for biodiversity, are increasingly at risk of being eroded, which may undermine conservation efforts in the long run (Aswani et al. 2018).

### Curbing and controlling the commercial trade of tropical wildlife

Demand reduction for illegal wildlife products is an essential component in curbing unsustainable hunting across many tropical regions, especially in places like Southeast Asia where a highly commercialized wild meat industry has created an insatiable demand for wildlife products from tropical forests around the world (Drury 2011; Gray et al. 2018). Official data on the illegal wildlife trade often stem from confiscations and are likely the tip of the iceberg, thus severely underestimating the quantities of commercialized wildlife (Sas-Rolfes et al. 2019).

Reducing consumer demand will require collaborations on international, national, and local levels, and must incorporate education and awareness campaigns that simultaneously work toward shifting cultural norms as well as address weak institutional capacity to control and enforce wildlife trade bans. Both would benefit from more interdisciplinary approaches. To date, many public demand reduction campaigns focused on voluntary behavioral change as it relates to illegal wildlife trade have not utilized the full spectrum of knowledge present within the social sciences. Such programs could benefit, for example, from the adoption of strategic behavioral change approaches that are based on key societal pressures that make illegal wildlife socially unacceptable (Wallen and Daut 2018). In situations where demand for wild meat is linked to extreme poverty (de Merode et al. 2004), outreach campaigns may need to be coupled with economic incentives to reduce reliance on forest products—though it should be noted that, in some tropical countries, demand for wildlife products is not primarily poverty-driven (Nguyen and Roberts 2020), in which case such economic incentives would not be appropriate. Furthermore, countries from the Global North and international coalitions have a responsibility to help strengthen local capacities so that tropical countries are better able to control illegal wildlife trade within their own borders. The gap between global and local environmental governance is well known but, as pointed out by Sas-Rolfes et al. (2019), this topic has not been discussed often within the specific context of reducing illegal wildlife trade. Altogether, without substantial progress in demand reduction, the magnitude and pace of tropical defaunation will continue to increase, undermining multiple components of SDG 15 (target 15.5, 15.7, 15.c) and heightening the threat of wildlife-borne communicable diseases spreading to humans (SDG 3, target 3.3).

### Including fauna in forest focused global climate finance

The SDGs include specific targets that provide a chance to support the mitigation of defaunation. SDG 13 is a particularly relevant example since climate change is expected to become a major driver of global biodiversity loss; for instance, SDG 13 target 13.A—mobilizing jointly $100 thousand million annually by 2020 from all sources to address the needs of developing countries in the context of meaningful mitigation actions. In the context of forest rich developing countries this includes forest-based climate mitigation, for example through implementing REDD+ mechanisms (Bastos Lima et al. 2017). It is imperative that global forest governance protects ecological processes instead of focusing solely on maintaining forest cover, as is currently done by most well-established approaches, such as REDD+ (Krause and Nielsen 2019). A holistic ecological and socio-ecological perspective that includes preserving healthy faunal communities is fundamental to maintaining intact tropical ecosystems. Monitoring forest cover using remote sensing as a simple metric of conservation success is therefore insufficient to understanding the ecological degradation that occurs under tropical forest canopies as a result of defaunation. Thus, in order to assess the success of forest conservation it is imperative to work with local communities and forest users whose activities affect forest fauna, for instance through participatory forest monitoring that includes regular fauna inventories (Krause and Zambonino 2013).

In defaunated but otherwise intact forests, the re-introduction of key species might be an option to reverse defaunation and therefore restore ecological processes (Galetti et al. 2017; Sobral-Souza et al. 2017), provided that appropriate local community support and participatory monitoring and management is ensured (Krause and Zambonino 2013). Indeed, given the magnitude of defaunation that has already taken place in the world’s tropical forests (Benítez-López et al. 2017), re-wilding may be fundamental to the successful restoration of degraded forests (target 15.2) and their functioning as carbon sinks in the long-term (target 13.1).

### Recognizing trade-offs, but not at the cost of local communities

Fundamentally, policy-makers, forest managers, and local communities alike must realize that forest fauna is the very foundation of a large range of ecological functions that underly nature’s contribution to people from the local to the global level. The provision of food through pollination and seed dispersal (SDG 2), the regulation of disease vectors and cultural and spiritual significance (SDG 3), the contribution to biomass carbon storage (SDG 13), and the preservation of terrestrial biodiversity (SDG 15) are just some of the important benefits of functional diversity in tropical forests.

Nonetheless, we must reject blaming biodiversity loss on groups of people who rely on forests for their daily livelihoods, and there is a need to rethink narratives that resulted in the stigmatization of hunters and the consumption of wild meat, particularly for subsistence purposes (van Vliet 2018). In some situations, the narrative of a ‘bushmeat crisis’ will not help to garner local support in developing more sustainable wildlife management strategies. Instead, conservation and forest governance must engage with those who sustainably hunt and harvest forest fauna as their allies for an effective and equitable long-term sustainable management of forests.

### Understanding and tackling the globality of biodiversity loss

Lastly, any long-term effective approach to protect tropical forest fauna—and, broadly, global biodiversity itself—depends on how we understand and address drivers that are beyond the local or national level. Global commodity chains and the international demand for agricultural products, minerals, and forest resources have implications for the survival of numerous species (Moran and Kanemoto 2017) because they drive habitat alteration and the harvesting and hunting of forest fauna for international trade (Harrison et al. 2016). Intergovernmental Science-Policy Platform on Biodiversity and Ecosystem Services (IPBES) has, as one of the first global policy relevant bodies, recognized and explicitly pointed out the global indirect drivers of biodiversity loss (IPBES 2019). The next Conference of the Parties to the Convention of Biological Diversity (CBD), scheduled for 2021, must follow suit and can no longer blind itself to the many threats the global economic systems pose to the survival of wildlife around the world. Setting politically acceptable targets that are repeatedly missed by the international community is unacceptable and a threat not just for biodiversity but for human life and well-being around the world.

## Conclusion

There is a great need to clearly communicate the role of fauna in maintaining the ecological functions of tropical forests. It is not enough to focus on tree cover in forest governance and forest protection alone, but the drivers of defaunation must be understood and addressed as well if these ecosystems are to provide the services humans clearly benefit from. We have described how defaunation is interlinked with sustainable development, using the SDG framework. Although we selected only four of the 17 SDGs, the existence of additional interlinkages between other SDGs and biodiversity at large, and defaunation in particular, should not be disregarded.

We briefly described the numerous consequences of defaunation. What happens under the canopy of the world’s remaining tropical forests is of the utmost importance for achieving the SGDs. An empty forest is inherently different than a forest with a healthy faunal community. Moreover, for everything that we know about the myriad consequences of tropical defaunation, there is almost certainly much more that we do not know, due to the fact that research on defaunation is still in its nascent stages, the complex and still-poorly understood implications of perturbing tropical ecosystems, and the long time periods over which defaunation impacts are likely to play out. The true consequences of tropical defaunation for global biodiversity and humanity therefore remain unknown—but are almost certainly underestimated. Given the multi-faceted and profound consequences of ongoing tropical defaunation, it is imperative that defaunation receives the same level of attention that has been given to other major threats to humanity, such as the most recent global response to the SARS-CoV-2 pandemic.

The demand for wild meat, wild animal parts, and other forest resources represents a Damocles sword, putting at risk the ecological functions of forest ecosystems and, ultimately, threatening the well-being of local communities and global society. However, at the same time blanket bans on wildlife consumption and hunting implies adverse effects for those people who depend on these as an important income and food source. Tropical forest fauna can be a renewable resource if used sustainably. Preventing defaunation is crucial for maintaining ecological functions and evolutionary relationships that support biodiversity and carbon storage, for the intrinsic value that societies and cultures place on biodiversity, and ultimately for people and future generations across the world.
